# Editorialets

**Published:** 1910-11

**Authors:** 


### Editorialets.

STATE HEALTH OFFICER BRUMBY has withdrawn his resignation and will serve his term out—to January 15, the inauguration of the new administration.

---

DR. FLY'S EXCELLENT PAPER in this issue will be read with interest. He is well qualified to speak on the important subject, having had large experience both as a surgeon, one of the most distinguished in the South, as mayor of Galveston and now as chairman of the Hospital and Medical College Committee of the Board of Regents of the University of Texas.

WANTED.—A graduated and licensed physician, especially experienced in physical therapeutics, including electric and hydrotherapy, and expert in the use of the appliances for such treatment, wishes a position as such in a sanatorium, or with a physician needing such assistant. Address Dr. H. C., care this JOURNAL.

---

VERDICT in "The Strange Case of Dr. Bruno":

"I couldn't put it down."—*Everybody.*

"It is indeed a remarkable book."—*Lancet-Clinic.*

"Jules Verne nor Robert L. Stephenson ever took loftier flights of fancy."—*Statesman.*

$1.50; with the "Red Back" one year, $2.

---

BROOKVILLE, IND., October 22, 1910.

OCTOBER number of the "Red Back" received and read with interest. I have read many medical magazines but this number is the *best single issue* I have ever seen. The psychologic treatment of the inebriety question (Williams), the plain matter of fact account of hookworm infection (Bass), and your own superb paper (Telepathy and Telegraphy)—three premiers in one number.

C. HENRI BOGART.

BUFFALO, TEXAS, October 20, 1910.

MY DEAR DOCTOR DANIEL: Enclosed find one of Uncle Sam's notes for $1 for which you will move up my subscription to the *"one only"* "Red Back." May its back never fade nor its pages cease to dispense their peerless scintillations in defense of truth, justice and a square deal.

Sincerely your friend,

SAM R. BURROUGHS.

[Twenty-six consecutive years.—D.]

---

TAYLOR-LAKE.—Cards received announcing the marriage, at Marshall, Texas, October 5, of Dr. Holman Taylor, Secretary Texas State Medical Association and editor of its journal, to Miss Frances Eleanor Lake, of Marshall. I have not the honor of Miss Lake's acquaintance, but I surely congratulate her on the capture of our bright, handsome and popular Editor-Secretary. Long life and happiness to them.

---

PACHYDERMATOUS DOCTORS.—It is a strange phase in the character of some doctors that while they are seemingly sensitive to criticism as to professional ethics, and wish to be thought well of, they are thick-skinned, and really reckless of their credit, dishonest, in fact. I have on my books scores of thought-to-be reputable doctors, some of high standing at medical meetings, who take prominent part in the proceedings, who do not mind refusing a small draft or bill for an honest debt any more than another kind of "duck" minds a shower. They are insensible to any kind of appeal. I have a list of them, my D. B. list, and sometimes big insurance companies ask me about medical examiners. Are you on the list, doctor? How many bills have you chucked in the waste basket this year?

---

Brag is a Good Dog, Hold-Fast is a Better.—Referring to the above. Look first on that picture, then on this:

Renewing for twenty-sixth conservative year, Sharp & Dohme write: "If we could advertise in only two medical journals in the U. S., the 'Red Back' would be one of them."

Dr. J. W. Kenney, Sanatorium, San Antonio, Texas, writes: "My $4.50 ad in February number brought me more than as many hundred dollars."

Parke, Davis & Co., Fairchild Bros. & Foster, Fellows, Lambert, Phillips, Battle, Arthur-Peters Co., Mellier—oh, hosts of the best advertisers have been in the "Red Back" from twenty to twenty-five years.

---

Dr. J. H. Kellogg of the famous Battle Creek Sanitarium has recently visited Southern Texas, and at Austin and San Antonio he delivered a lecture on "The New Hygiene," and "Are We a Dying Race?" These lectures, the latter beautifully and copiously illustrated by stereopticon, were under the auspices of the Congress of Mothers, then in session at Austin. The great auditorium of the Texas University was packed with a splendid audience to hear him, and they were simply delighted. Dr. Kellogg is a wonder—one of the most delightful men to meet. He advocates dietic reform as essential to health, and made many converts to a simpler diet and a "back to nature" life.

---

Dr. W. P. Powell.—The editor had the happiness to meet his very dear and loyal old friend, Dr. Powell, of Willis, Texas, in Austin last week at the unveiling and dedication of the monument erected to the memory of the immortal Hood's Brigade. Our acquaintance began at Belton in 1884, since which time he has been a most devoted and greatly loved friend. He is old and infirm now, but, socially, is as cheery, joyous, lovable and delightful as then. He was a member of Hood's Brigade, and was badly wounded at second Manassas and was later Assistant Surgeon of the Fifth Texas; participated in battle of Gettysburg; was taken prisoner there, and left in charge of three hundred wounded. Oh, if all men were like dear old Dr. Powell, and Swearingen and Cupples, what a delight it would be to live in communion with our fellowman.

---

A WAIL OF DESPAIR.—Jonesey-the-Joke, of the California Tentacle of the Chicago Octopus, cries: "Help us, cash us or we sink." The following is from the leading editorial in his October number: "*This Is Disheartening.*—How in the world do you suppose an advertising committee or a publication committee or an editor can continue forever to devote a great deal of time and energy to building up your journal when you, as individuals, will not do much if anything to help? *Some time ago, after a good deal of persuasion, we induced an Eastern manufacturer to place a half-page advertisement in the journal for four months, assuring him that the members of the society took a good deal of interest in the advertising pages of their publication.** The preparation advertised was, of course, approved by the Council on Pharmacy and Chemistry, and in the advertisement the manufacturer offered to send samples or literature on request. At the expiration of the four months he wrote, saying, *"We are sorry to say that we did not receive one single inquiry relating to your journal."*

---

IS THE MEDICAL BRANCH of the University thoroughly equipped for biological, chemical and pathological research as reported by Flexner, and the equal of Johns Hopkins and other schools? We shall see. I have been called down by an eminent physician of Galveston, once connected with the Medical Department of the University, and who does not wish to be named, and assured that certain statements in my editorial in October issue, *"Much Ado About Nothing,"* are incorrect. He claims that the Medical Branch *is* thoroughly equipped for teaching all branches—as stated by Flexner and denied by yours truly. That denial I still contend for and can prove it; but he says that my statement that "One teacher after another, after a brief stay, more or less, resigned because of inadequate research facilities," is not correct; that Prof.

*Italics are mine.—Daniel.

Smith resigned to take a chair and increased salary in his alma mater, the University of Pennsylvania; Thayer to take a position in a sanatorium somewhere, and that Dock never taught in the Medical Branch, but in the Galveston Medical College before the Medical Department of the University of Texas was organized. All this is conceded. I was wrong. But the fact remains that Austin (chemist and not pathologist as stated by me) "couldn't work without tools," as stated, and rather than do so he resigned a $3000 position for one at a salary of $2000. As to the equipment, credited by Flexner, who puts Texas in the top row along with Johns Hopkins and only four others, if such were the case why should the Regents have besought the Legislature on their knees, as one member of the Regents expressed it, to give the University money to provide better facilities? The Legislature appropriated $100,000 for better research facilities and suitable buildings in order that the students working in the alleged chemical laboratory in the basement of the college should not be compelled—as they were, after a rain—to stand on bricks or soap boxes to get up out of the mud and water while using the microscope? This and the foregoing statement is made upon the authority of and with permission to use, by Dr. A. W. Fly of the Board of Regents. Mr. Campbell, in pursuance of his policy (?) of crippling the fundamental interests of the State, the public health and education, vetoed the appropriation, thus sacrificing those dearest interests to his narrow notions of "economy." He should be given a leather medal and Flexner another.

"Which is why I remark, and my language is plain"—the Medical Department of the University is *not* adequately equipped for research work—necessary to a thorough education in medicine!

